# LEGO-lipophosphonoxins: length of hydrophobic module affects permeabilizing activity in target membranes of different phospholipid composition†

**DOI:** 10.1039/d3ra07251g

**Published:** 2024-01-17

**Authors:** Milica Dugić, Hana Brzobohatá, Viktor Mojr, Tereza Dolejšová, Petra Lišková, Duy Dinh Do Pham, Dominik Rejman, Gabriela Mikušová, Radovan Fišer

**Affiliations:** a Department of Genetics and Microbiology, Faculty of Science, Charles University Viničná 5 128 00 Prague 2 Czech Republic fiserr@natur.cuni.cz; b Institute of Organic Chemistry and Biochemistry, Czech Academy of Sciences v. v. i. Flemingovo nám. 2 166 10 Prague 6 Czech Republic

## Abstract

In the past few decades, society has faced rapid development and spreading of antimicrobial resistance due to antibiotic misuse and overuse and the immense adaptability of bacteria. Difficulties in obtaining effective antimicrobial molecules from natural sources challenged scientists to develop synthetic molecules with antimicrobial effect. We developed modular molecules named LEGO-Lipophosphonoxins (LEGO-LPPO) capable of inducing cytoplasmic membrane perforation. In this structure–activity relationship study we focused on the role of the LEGO-LPPO hydrophobic module directing the molecule insertion into the cytoplasmic membrane. We selected three LEGO-LPPO molecules named C9, C8 and C7 differing in the length of their hydrophobic chain and consisting of an alkenyl group containing one double bond. The molecule with the long hydrophobic chain (C9) was shown to be the most effective with the lowest MIC and highest perforation rate both *in vivo* and *in vitro*. We observed high antimicrobial activity against both G^+^ and G^−^ bacteria with significant differences in LEGO-LPPOs mechanism of action on these two cell types. We observed a highly cooperative mechanism of LEGO-LPPO action on G^−^ bacteria as well as on liposomes resembling G^−^ bacteria. LEGO-LPPO action on G^−^ bacteria was significantly slower compared to G^+^ bacteria suggesting the role of the outer membrane in affecting the LEGO-LPPOs perforation rate. This notion was supported by the higher sensitivity of the *E. coli* strain with a compromised outer membrane. Finally, we noted that the composition of the cytoplasmic membrane affects the activity of LEGO-LPPOs since the presence of phosphatidylethanolamine increases their membrane disrupting activity.

## Abstract 2

We developed modular antimicrobial compounds capable of inducing cytoplasmic membrane perforation. This structure–activity relationship study focuses on the role of the length of their alkenyl hydrophobic module.

## Graphical abstract

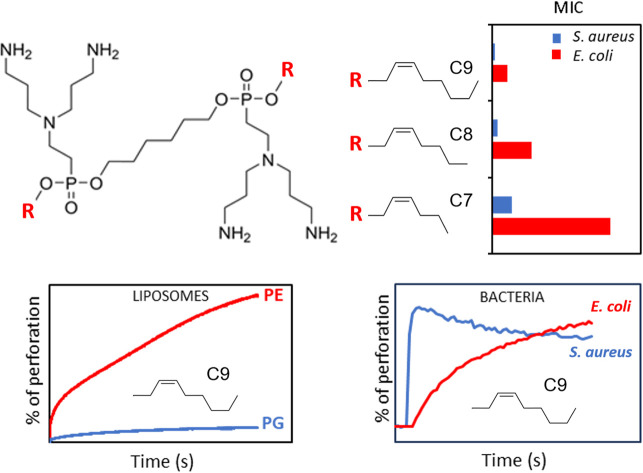

## Introduction

Natural antimicrobial peptides (AMPs) are small cationic molecules that are often referred to as host defense peptides thanks to their protective properties against a wide variety of bacterial, viral, fungal, and protozoan infections.^1–4^ These molecules exert great structural variety. In addition to their positive charge, other characteristics such as size, primary sequence, conformation, hydrophobicity and amphiphilicity play an important role in their antimicrobial activity.^5,6^ Understanding of AMP mode of action facilitates the design of new, effective antimicrobial peptides^4^ which is of a great importance due to an emerging bacterial resistance to practically all known antibiotics and spreading the resistance in a population of bacteria.^7,8^

So far, nearly all the proposed mechanisms of AMP action include weak electrostatic interactions with membrane lipids followed by hydrophobic interactions leading to bacterial membrane perforation. Key for this interaction is the composition and overall charge of the cell membrane that significantly differs between eukaryotic and prokaryotic cells.^7,9^ Eukaryotic membrane lipids are mainly neutral glycerophospholipids such as phosphatidylcholine and sphingomyelin while the bacterial cell membrane is essentially composed of negatively charged lipids, such as phosphatidylglycerol (PG), cardiolipin, and the zwitterionic phosphatidylethanolamine (PE). Because of that, lower affinity of cationic AMPs towards eukaryotic cells is to be expected.^7,10^ Resistance development of a sensitive strain to AMPs is of low probability due to the severe changes in the bacterial membrane structure needed to establish effective resistance.^1^ It is highly probable that these changes would impair proper membrane function.

Design of novel antibiotic molecules is focused on mimicking the desirable features of natural AMPs like their charge, hydrophobicity and amphiphilicity. Even though it seems generally easy to design new AMPs by combining eligible features together, the *in vivo* stability, low solubility and high cytotoxicity can present an insuperable obstacle,^11^ together with the difficult manufacturing.^12^ On the other hand, these issues stimulated the development of synthetic antimicrobial peptides (SAMPs) with various advantages, such as potent activity, low production cost and reduced toxicity.^13,14^ Rational design of SAMPs is the key to making a molecule with the desirable activity. As mentioned above, electrostatic and hydrophobic interactions are essential for the antimicrobial activity of AMPs allowing peptides to interact and penetrate the membrane. However, high hydrophobicity can lead to unwanted features like increased hemolytic activity, while charge variations can affect SAMP-cell membrane interaction.^15^ Too polar compounds can interact with low affinity with bacterial membranes while too hydrophobic compounds can fail to discriminate between eukaryotic and prokaryotic target membranes.^16^ Because of that, the relationship between molecule structure and function must be carefully established during SAMP design.^13,15^

One of the SAMPs with significant antimicrobial activities are compounds termed lipophosphonoxins (LPPO) which we have designed and synthesized. LPPOs are synthetic small molecules that exhibit considerable antibacterial activity against a broad spectrum of bacteria, including multidrug-resistant strains, without cytotoxicity on human cells at bactericidal concentrations^17–19^ and low propensity to develop bacterial resistance. Our studies have demonstrated that LPPOs act by permeabilizing the bacterial membrane, leading to its disruption and cell death. So far, we have synthesized and tested three generations of LPPO. The first generation (LPPO I) shows activity against G^+^ bacteria with moderate selectivity. It consists of a hydrophilic moiety with a small positive charge as the polar module (PM), a linear alkyl chain (C14-16) as the hydrophobic module (HM), and nucleoside uridine as the auxiliary module (NM), all the parts connected using connector module (CM).^17,18^ The second generation LPPO (LPPO II) exhibits activity against both G^+^ and G^−^ bacteria with moderate selectivity. In this generation, the PM is a hydrophilic moiety with an increased positive charge.^19,20^ LPPO II has already been successfully evaluated as an antibacterial additive in the bone cement^21^ and as a wound dressings based on the polycaprolactone nanofiber scaffold (NANO) releasing second generation lipophosphonoxin (LPPO) as the antibacterial agent.^22^ Major structural alteration led to the newest generation called LEGO-LPPO,^23^ which demonstrated activity against both G^+^ and G^−^ bacteria, and improved selectivity. Moreover, their activity is not influenced by the presence of serum albumins that presented complications in previous LPPO generations.^23^

**Fig. 1:**
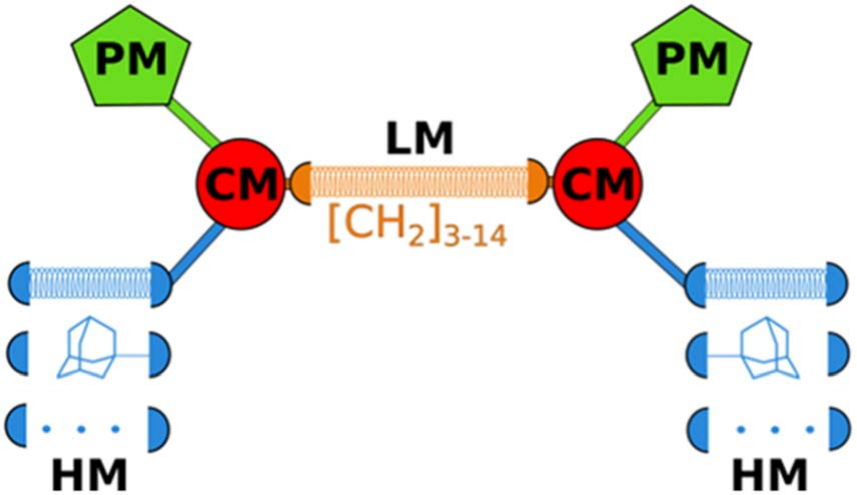
General structure of LEGO-LPPO (PM = polar module, CM = connector module, HM = hydrophobic module, LM = linker module). The coiled springs indicate variable length or different modules.

**Fig. 2:**
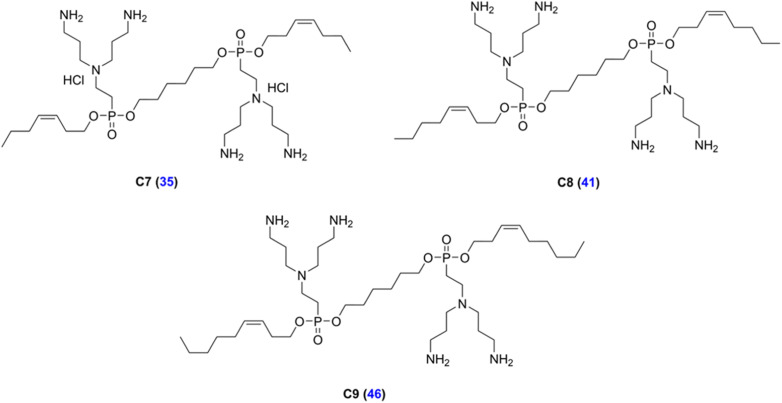
Structures of the LEGO-LPPOs selected for the study (C7, C8, C9). Blue numbers in parentheses represent compound numbers from Do Pham *et al.*, (2022).^23^ The structure of C10 is presented in ESI Fig. S1.†

LEGO-LPPO are based on a general symmetrical structure (depicted in Fig. 1) composed of two hydrophobic modules (HM), two polar modules (PM), two connector modules (CM), and a linker module (LM). The key feature of the molecule is the hydrophobic module which plays a critical role in insertion of the molecule into the bacterial cytoplasmic membrane. Thus, among others we prepared a series of LEGO-LPPO molecules differing in the length of the hydrophobic alkenyl chain.^23^ Based on the lowest possible MIC and the highest possible HC_50_ we further selected closely related compounds – these are LEGO-LPPOs with bis(3-aminopropyl)amino groups as PM, linear six CH_2_– long LM, phosphonoethyl groups as CM, and hept-3-enyl (C7), oct-3-enyl (C8), non-3-enyl (C9) and dec-4-enyl (C10) groups (all in cis configuration) as HMs (Fig. 2 and S1†). Further in the text these molecules will be designated as C7, C8, C9 and C10, based on the number of carbon atoms in the alkenyl hydrophobic module. The comparison of antimicrobial activity of all previously published LEGO-LPPO and selected standard antibiotics on *S. aureus* and *E. coli* is presented in ESI Fig. S2.†

In this research, our aim was to explore structure–activity relationships of LEGO-LPPO molecules differing in the lengths of hydrophobic module (Fig. 2). We used both living bacteria and model lipid bilayers mimicking cytoplasmic membranes of the tested bacteria. We wanted to uncover how the length of the hydrophobic module affects LEGO-LPPO antimicrobial activity, pore formation and the mechanism of membrane permeabilization.

## Materials and methods

### Compounds selected for the study

Experiments in this study were performed with LEGO-LPPOs whose synthesis has been described previously.^23^ LEGO-LPPOs used in this research were compounds 35, 41, 46, and 48 according to Do Pham *et al.*, (2022)^23^ (designated here as C7, C8, C9, and C10, respectively) differing in the length of the alkenyl hydrophobic module, by one carbon atom (Fig. 2 and S1†).

### Bacterial strains

We used strains of G^+^ (*Staphylococcus aureus* CCM 4223, *Bacillus subtilis* 168 trp^+^ BaSysBio) and G^−^ (*Escherichia coli* CCM 3954) bacteria obtained from the Czech Collection of Microorganisms (CCM), Faculty of Science, Masaryk University Brno. To study the role of the outer membrane (OM) in LEGO-LPPO action, *E. coli* imp4213 was used. This strain has an in-frame deletion of *lptD* gene encoding LptD protein, important for the LPS assembly in OM.^24^

### Determination of minimum inhibitory concentrations (MIC)

MIC of the molecules C7, C8, C9, and C10 was determined in a disposable microtitration plate by standard microdilution method.^25^ LEGO-LPPOs were diluted in Mueller–Hinton medium (Mueller–Hinton, BioRad, France) to set the concentration in range of 0.125–16 mg L^−1^. Plates were then inoculated with the standard number of tested bacteria (10^5^ CFU mL^−1^ in each well). MIC was read after the overnight incubation at 37 °C. To check the presence of surviving bacteria under inhibiting concentrations, 5 μL of cell suspension from each well was inoculated onto Mueller–Hinton agar plates. After incubation for 24 hours at 37 °C the Minimum Bactericidal Concentration (MBC) was determined as the lowest concentration that showed no visible growth of colonies on the agar plates.

### Determination of hemolytic activity

The hemolytic activity of LEGO-LPPO was determined as the amount of hemoglobin released from the red blood cells (RBCs). The blood was aseptically collected from a volunteer human donor using an acquisition protocol adhered to the requirements of the Ethics Commission of the Charles University. The volunteer had signed the informed consent. The pellet was harvested from collected blood after centrifugation (1000 g, 4 °C, 10 min, Micro 220R, Hettich), supernatant was discarded, and RBCs were washed three times and resuspended in 150 mM NaCl to the final concentration of 2% (v/v). Aliquots were mixed with the tested compound (final concentration 5–200 mg L^−1^) as well as with positive (Triton X-100 1%, v/v) and negative (150 mM NaCl) control and incubated for 3 h at 37 °C. After incubation, the mixture was centrifuged (1000 g, 4 °C, 10 min) and the supernatant (300 μL) was transferred to 1700 μL of 1% Triton X-100 dissolved in distilled water. Absorbance was measured at 540 nm on the spectrophotometer (Beckman DU530). The hemolytic activity was expressed as the concentration of tested compound that caused lysis of 50% RBCs (HC_50_) when compared to 100% hemolytic activity of positive control (Triton X-100). In the case of low hemolytic activity (HC_50_ > 200 mg L^−1^), the linear extrapolation was used. The experiments were performed as three independent examinations with three replicates for each sample. Data were expressed as means of HC_50_ ± S.D.

### Membrane permeabilization assay

Bacterial cultures were aerobically grown with shaking (37 °C, 180 rpm) in LB medium to mid-log phase (OD_450_ = 0.5), harvested (8000 g, 25 °C, 10 min), washed, and resuspended (final OD_450_ = 0.2) in a buffer containing 10 mM HEPES (pH 7.2), 0.5% glucose, and 10 μM Propidium Iodide (PI, Invitrogen). Cell suspension was then pipetted into a pre-prepared black 96 well plate (BRAND*plates* pureGrade) with the concentration range of LEGO-LPPOs in triplicates. PI uptake into cells, its incorporation to DNA and the increase of its fluorescence indicates membrane permeabilization. Permeabilization effect was monitored as the fluorescence intensity (excitation at 515 nm, emission at 620 nm, bandpass 5 nm) at 25 °C using a FluoroMax-3 spectrofluorometer with MicroMax plate reader (Jobin Yvon, Horiba). Optical filters 3RD500-530 and 3RD570LP (Omega Optical) were used in excitation and emission paths respectively, for suppression of light scattered by the cells. Negative controls were non-treated cells, while 5 μM melittin (Sigma) was added as a positive control of cell permeabilization. Representative kinetics from at least two independent experiments performed in triplicates are shown. The rate of relative membrane permeabilization (% min^−1^) was calculated from the kinetics as the first derivative and fitted with an exponential function (initial phase, time 0–40 min) and a Gaussian function (delayed phase, time ∼20–120 min) using Fityk software.^26^ Note that LEGO-LPPO concentrations effective in PI assay do not respond to MIC due to the different number of cells necessary for reaching detectable signal during the measurement. During the MIC determination, we used 10^5^ CFU mL^−1^, while for PI measurements 10^7^ CFU mL^−1^ was used.

### Liposome preparation

For liposome preparation we used 1,2-dioleoyl-*sn*-glycero-3-phospho-(1′-rac-glycerol) (DOPG), 1,2-dioleoyl-*sn*-glycero-3-phosphoethanolamine (DOPE) and 1,1′2,2′-tetraoleyl cardiolipin (TOCL) purchased from Avanti Polar Lipids. Lipids were dissolved in chloroform and appropriate amounts were taken for a lipid film formation. Lipid films with compositions mimicking G^+^ bacteria (DOPG : DOPE 2 : 1, w/w representing *B. subtilis* and DOPG : TOCL 2 : 1, w/w representing *S. aureus*), and G^−^ bacteria (DOPE : DOPG 2 : 1, w/w representing *E. coli*) were prepared on the wall of a glass tube. The solvent was evaporated under a nitrogen stream to obtain a thin layer of lipids (the evaporation under vacuum gave comparable results in terms of LEGO-LPPO activity and the influence of liposome composition). Inner buffer containing carboxyfluorescein (CF, 50 mM 5(6)-carboxyfluorescein, 5 mM HEPES, pH 7.4) or ANTS/DPX (15 mM 8-aminonaphthalene-1,3,6-trisulfonic acid, 30 mM *p*-xylene-bis-pyridinium bromide, 100 mM NaCl, 50 mM Tris, pH 7.4) was added to the dry lipid film and the tube was shaken for 60 min to form multilamellar vesicles. Multilamellar vesicles were then repeatedly extruded through polycarbonate filters (pore size 100 or 1000 nm, Whatman) using an extruder (Avanti Polar Lipids) to obtain large unilamellar vesicles (LUV_100_ or LUV_1000_) of uniform size (for details of the liposome size see respective figure legends). Liposomes were then separated from non-encapsulated dye by gel filtration on Sephadex G-50 using an elution buffer A (for carboxyfluorescein loaded vesicles: 100 mM NaCl, 0.5 mM Na_2_EDTA and 5 mM HEPES, pH 7.4) or elution buffer B (for ANTS/DPX loaded vesicle: 200 mM NaCl, 50 mM Tris, pH 7.4). The quality of liposomes was tested by DLS (ESI Fig. S3†). Concentrated liposome suspension was diluted in the elution buffer to a final concentration of 10 μM (according to the assessed content of inorganic phosphate).^27^

### Carboxyfluorescein leakage from liposomes

LUV_1000_ of a final concentration of 10 μM in 2 mL volume were treated with a range of LEGO-LPPO concentrations (0.1–12.5 mg L^−1^). The increase of fluorescence intensity (*F*, excitation 480 nm, emission 515 nm, band paths 2 nm) induced by leakage of CF content from the liposomes was recorded over time and normalized to the maximum intensity (*F*_max_) induced by lysing the vesicles with 0.1% Triton X-100. CF leakage was quantified using the following equation: % CF leakage = (*F* − *F*_0_)/(*F*_max_ − *F*_0_) × 100% where *F* is an actual fluorescence intensity, *F*_0_ is the fluorescence intensity before the addition of permeabilizing agent and *F*_max_ is the maximum fluorescence caused by complete disruption of all liposomes by Triton X-100.

### LEGO-LPPO cooperativity – Hill function

To estimate the cooperativity of the LUV leakage (or permeabilization of bacteria for PI) induced by LEGO-LPPO, we fitted the data with the Hill function:*Θ* = [C]/((*K*_A_)^*n*^ + [C]^*n*^) × 100%where *Θ* is the observed leakage (%), [C] is the LEGO-LPPO concentration, *K*_A_ is the concentration of LEGO-LPPO producing the half-maximal effect, and *n* is the Hill coefficient.

### Laurdan generalized polarization (GP)

The stock suspension of 100 μM LUV_100_ was labeled with 0.2 μM concentration of 2-dimethylamino-6-lauroyl naphthalene (Laurdan, Molecular Probes, dissolved in dimethylsulfoxide, DMSO) at 37 °C for 60 min, the final ratio of Laurdan per lipid was 1 : 500. Final concentration of DMSO was 0.2% in all samples. The LUV suspension was split to individual aliquots of 0.1 mL. To study the possible shift in packing of the lipids in membranes with two different compositions (DOPE : DOPG and DOPG : DOPE, 2 : 1, w/w), we measured the generalized polarization (GP) of Laurdan-labeled LUVs under stable temperature 27 °C using FluoroMax-3 spectrofluorometer (Jobin Yvon, Horiba). Fluorescence spectra were measured from 400 to 600 nm (after excitation at 365 nm), with 4 nm bandpass. GP values were calculated according to Parasassi *et al.* (1990).^28^

## Results

### Determination of minimum inhibitory concentrations (MIC) and hemolytic activity (HC_50_)

Same as the previous LPPO generations, the dominating LEGO-LPPO mode of action is pore formation.^23^ In this study, we focused on the LEGO-LPPO hydrophobic module represented by an alkenyl group (alkyl chain with a double bond) (Fig. 2). We aimed at finding out how the alkenyl chain length affects LEGO-LPPO activity in G^+^ and G^−^ bacteria evaluated as MIC and cytotoxicity by determining their hemolytic activity against erythrocytes (HC_50_). We first tested a series of LEGO-LPPO molecules differing by one carbon atom in the chain length on selected bacterial strains, representatives of G^+^ and G^−^ bacteria.

The results in Table 1 show that LEGO-LPPO antimicrobial activity rises with the increasing length of the hydrophobic module from C7 up to C9. Molecules with longer hydrophobic module (HM) showed lower MIC apart from *B. subtilis* that showed comparably high sensitivity to all tested molecules. However, elongation of the alkenyl chain to C10 caused a decreased antimicrobial activity against *S. aureus*, *B. subtilis* and *E. coli* 3954 and it exerted considerable hemolytic activity (Table 1) at the same time. The substance C10 proved to be unusable for possible clinical application and we excluded it from further study.

**Table 1:** Minimum inhibitory concentration (MIC), hydrophobicity index (*c* log *D*) and hemolytic activity (HC_50_) of tested LEGO-LPPOs. The *c* log *D* values were taken from Do Pham *et al.*, (2022)^23^

Number of C atoms	MIC (mg L^−1^)				*c* log *D*	HC_50_ (mg L^−1^)
	*S. aureus*	*B. subtilis*	*E. coli* 3954	*E. coli* imp4213		
C7	4	1	24	4	1.28	325 ± 15
C8	1	1	8	2	1.52	221 ± 26
C9	0.5	1	3	2	1.76	321 ± 32
C10	2	2	6	2	1.93	24 ± 6

In general, G^+^ bacteria represented by *S. aureus* and *B. subtilis* appeared to be more sensitive to LEGO-LPPO action than G^−^*E. coli*. These results are in line with data which we published previously using a panel of G^+^ and G^−^ bacterial species.^23^ This implies that the outer membrane (OM) could present an obstacle for LEGO-LPPOs and that higher concentrations are necessary for the molecule to achieve growth inhibition. This hypothesis can be supported by higher sensitivity of *E. coli* imp4213 with a compromised OM when compared to *E. coli* 3954 (Table 1) with an intact OM, as discussed in the next section.

### Effect of outer membrane integrity and the HM length on LEGO-LPPO-induced membrane permeabilization (*E. coli*)

**Fig. 3:**
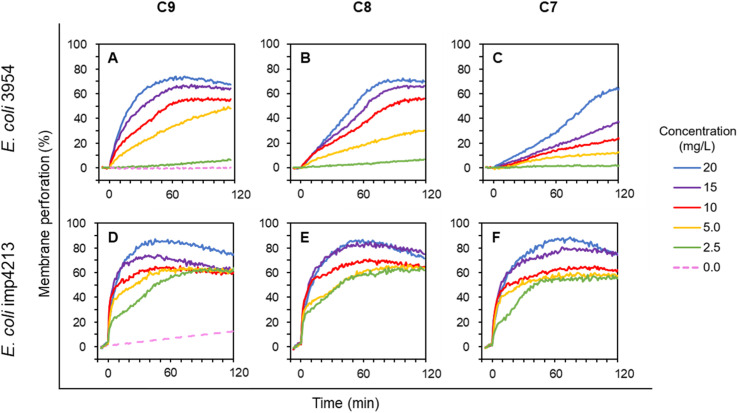
Membrane permeabilization of *E. coli* 3954 (A–C) and *E. coli* imp4213 (D–F, with impaired OM) by varying concentrations of LEGO-LPPOs (C9, C8, C7), added at time 0. Intensity of PI fluorescence indicates the probe influx across the permeabilized cytoplasmic membrane into the cells. Representative results from at least two experiments performed in duplicates are shown. See Materials and methods for details.

To further compare the activity of selected LEGO-LPPOs differing in the HM alkenyl chain lengths, we employed membrane permeabilization assay with living cells of *E. coli*. Propidium Iodide (PI) fluorescent dye used in this study enters the cells only after membrane damage. PI then binds to nucleic acid which leads to fluorescence intensity increase. Effectivity of membrane permeabilization induced by LEGO-LPPOs was measured in the concentration range of 1.25–20 mg L^−1^ on *E. coli* 3954 (Fig. 3A–C). The results show that membrane permeabilization was substantially affected by the length of the HM. Both the rate of the kinetics, especially in lower concentration, and maximum level of permeabilization changed with the HM length – the effectivity decreased in order C9 > C8 > C7. The C7 molecule with seven carbon atoms in HM showed the lowest perforation potential of *E. coli* 3954 and delayed action while C9 induced rapid action and permeabilization potential comparable to C8 when using the higher concentrations in the range of 10–20 mg L^−1^.

**Fig. 4:**
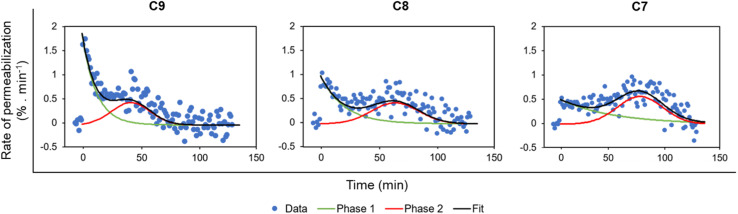
Rate of relative membrane permeabilization (% min^−1^) of *E. coli* 3954 by LEGO-LPPOs. The rates are derived from data shown in Fig. 3A–C (the concentrations inducing similar response were used: C9 10 mg L^−1^, C8 10 mg L^−1^, C7 20 mg L^−1^) and calculated as the first derivative of the measured curves. The data were fitted with an exponential function (phase 1, time 0–40 min) and a Gaussian function (delayed phase 2, time ∼20–120 min) using Fityk software.^26^ The two phases of membrane permeabilization are clearly distinguishable.

Permeabilization kinetics are not trivial but show a biphasic character (Fig. 4), especially in high concentration treatments (10–20 mg L^−1^). Within the first ∼30 minutes after LEGO-LPPO treatment, the initial phase takes place, possibly induced by LEGO-LPPO monomers or small complexes. The initial permeabilization rate is relatively high in the case of C9 and C8 but lower in case of C7. The second, still dramatic phase with high permeabilization rate starts approximately 40 minutes after the treatment (Fig. 3A–C and 4). This biphasic mode of permeabilization is more noticeable for the molecules with shorter HM (esp. C7) where the low permeabilization rate in the first phase produces kinetics of clearly sigmoidal character (Fig. 3C). We hypothesized that this manifestation might be caused by the presence of the bacterial outer membrane.

The outer membrane serves as a permeability barrier and can cause ineffectiveness of antimicrobial compounds in general. To test the hypothesis that OM affects LEGO-LPPO rapid action, we conducted an experiment with *E. coli* imp4213, a strain with deletion in the *lptD* gene coding for an essential OM protein. In complex with LptE it functions to assemble lipopolysaccharides at the surface of the OM.^24^ The *lptD* mutation (in *E. coli* imp4213) increases OM permeability making the cell sensitive to many different antibiotics and to bile salts.^29,30^ When using LEGO-LPPOs against this strain we observed a rapid action during the first minutes after addition (Fig. 3D–F). At high concentrations (10–20 mg L^−1^) the initial rapid LEGO-LPPOs action led to almost complete cytoplasmic membrane permeabilization. For lower doses (2.5–5.0 mg L^−1^) the concentration dependent initial rapid action was followed by a gradual permeabilization within ∼60 minutes after treatment. Major differences between the wild type and mutant *E. coli* strains were noted in the lowest treatment concentration (2.5 mg L^−1^), where LEGO-LPPOs showed much higher effectivity on *E. coli* imp4213 than on the *E. coli* 3954 under the same conditions. The difference was prominent within the first few minutes after LEGO-LPPOs addition (Fig. 3). The length of the HM seems to have more important role in efficiency of LEGO-LPPOs action against *E. coli* 3954 (with intact OM) than in the case of *E. coli* imp4213, since overall effectivity of the three LEGO-LPPO molecules was not markedly influenced by the different lengths of the HM on mutant strain (with the compromised OM) which was found to be overall more sensitive (see also Table 1).

### LEGO-LPPO-induced membrane permeabilization of G^+^ bacteria (*B. subtilis* and *S. aureus*)

**Fig. 5:**
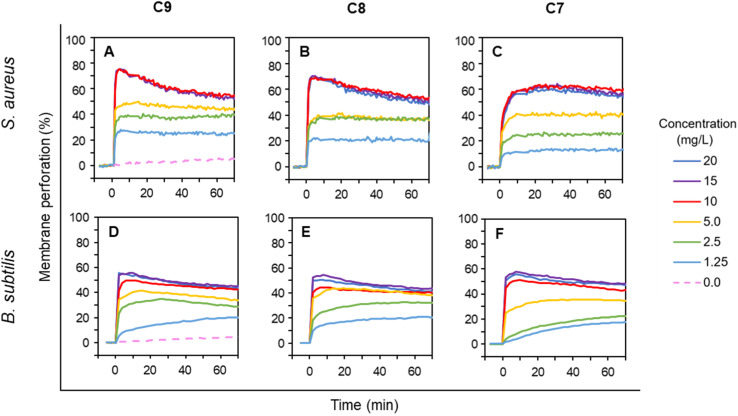
Membrane permeabilization of G^+^ bacteria *S. aureus* (A–C) and *B. subtilis* (D–F) by varying concentrations of LEGO-LPPOs (C9, C8, C7), added at time 0. Intensity of PI fluorescence indicates the probe influx across the permeabilized cytoplasmic membrane into the cells (see Materials and methods for details). Note that all the kinetics show the fastest permeabilization in the initial phase. Representative results from at least three experiments performed in duplicates are shown.

The experiments with G^−^*E. coli* strains suggested that the intact outer membrane hinders LEGO-LPPOs action when C9 is most effective and C7 is less effective in overcoming OM. Next, we wanted to explore how the presence of the outer membrane itself affects the LEGO-LPPO action on living bacteria. To directly detect the effects of LEGO-LPPOs on cytoplasmic membrane, we further tested LEGO-LPPOs action against selected G^+^ bacteria; *S. aureus* and *B. subtilis* in the range of concentrations 1.25–20 mg L^−1^. Rapid initial action of all LEGO-LPPOs molecules was noted for both G^+^ representatives (Fig. 5).

For C9 and C8 the membrane permeabilization of *S. aureus* reached the plateau within seconds after addition without any further intensity increase (Fig. 5A and B). For C7 at any tested concentration the initial fast action lasted about 5 minutes on *S. aureus* (Fig. 5C). The permeabilization of *B. subtilis* was continuous at lower concentrations but generally did not differ dramatically in respect to HM length, which seems to correspond to the MIC results (Table 1). The rapid initial action on G^+^ bacteria with absence of biphasic behavior observed on *E. coli* (Fig. 3 and 4) confirmed that the lack of OM allows LEGO-LPPOs to immediately reach the target and cause rapid membrane perforation.

**Fig. 6:**
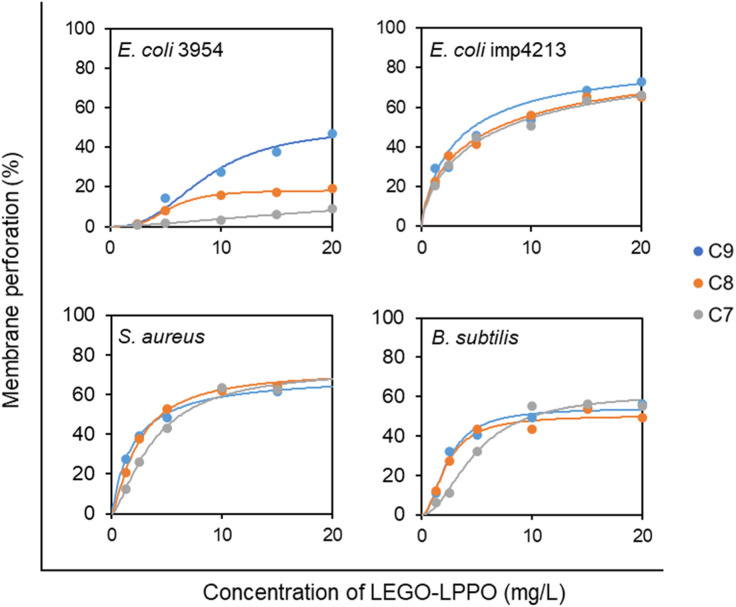
Concentration dependency of LEGO-LPPO activity against *E. coli* 3954, *E. coli* imp4213, *S. aureus*, and *B. subtilis*. Membrane perforation was quantified by PI assay after 20 min of incubation with LEGO-LPPOs (C9, C8, C7). Each point represents averaged normalized fluorescence intensity (*n* = 3) recorded in 96-well plates (the typical relative standard deviation was 5%). The curves show the best fit of the data with the Hill function (see ESI Table T1† for parameters values). The graphs include the data shown in Fig. 3 and 5.

Concentration dependency of LEGO-LPPO-induced membrane permeabilization (Fig. 6) shows similar trends on bacteria without OM (*B. subtilis* and *S. aureus*) and on *E. coli* imp4213 with compromised OM. On these strains the LEGO-LPPO activity (*K*_A_ values – concentration inducing half-maximal leakage, the final levels of membrane permeabilization and overall concentration dependency) depends slightly on the length of alkenyl group of the hydrophobic module (ESI Table T1†). A dramatic difference in efficiency among the LEGO-LPPO molecules was observed only in the case of *E. coli* 3954, where C7 clearly showed the lowest activity and C9 the highest activity.

### Effect of membrane composition on LEGO-LPPO activity (liposomes)

The differences in the overall course of permeabilization of LEGO-LPPOs on bacteria *in vivo* prompted us to test the extent to which the membrane phospholipid composition plays a role in LEGO-LPPO activity. For this purpose, we conducted *in vitro* experiments using carboxyfluorescein-loaded liposomes mimicking phospholipid composition of cytoplasmic membranes of individual bacteria. We prepared liposomes resembling *E. coli*, *B. subtilis* and *S. aureus* membranes in phospholipid ratios PE : PG 2 : 1, PG : PE 2 : 1 and PG : CL 2 : 1. These compositions roughly represent the most abundant lipids in the bacterial membrane of our selected strains.^31,32^

**Fig. 7:**
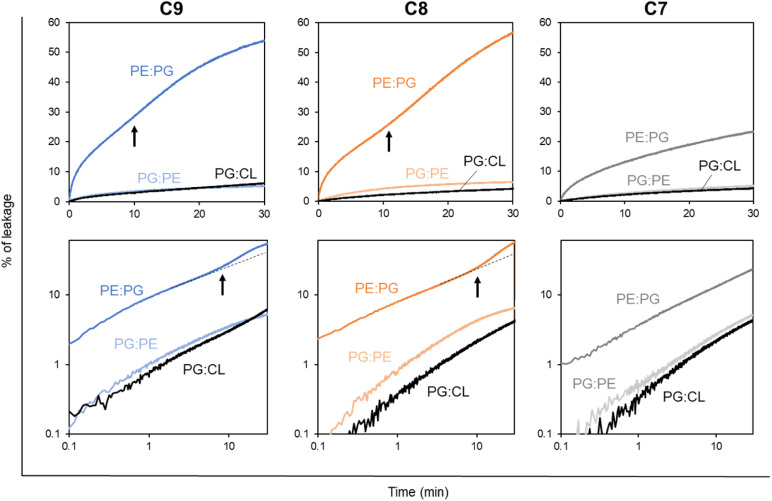
Leakage of CF-loaded liposomes of various membrane composition induced by LEGO-LPPO (C9, C8, C7 – see labels above graphs). Liposomes (LUV_1000_) composed of 2 : 1 mixtures – PE : PG (mimicking *E. coli*), PG : CL (mimicking *S. aureus*), PG : PE (mimicking *B. subtilis*) at 10 μM phospholipid concentration were treated by constant amount of particular LEGO-LPPO (1 mg L^−1^). The upper row of graphs shows the measured kinetics in the normal view, the bottom row of graphs shows the corresponding data in a double logarithmic plot. In a double logarithmic plot, a straight line (or a curve gradually bending downwards) can be expected for simple kinetics (single-hit). Black arrows point to the moment (∼10 min) which can be recognized as the onset of the second phase of lysis (kinetics curve upwards). The dashed black lines in the figure are guides for the eye. Clearly, all three tested molecules induce the highest leakage in PE : PG membranes. Note the biphasic character of the kinetics induced by C9 and C8 in PE : PG.

First, we measured the LUV leakage induced by LEGO-LPPOs in constant concentration of 1 mg L^−1^. Our results show (Fig. 7) the dramatic differences in LEGO-LPPO activity between G^−^ and G^+^ resembling phospholipid mixtures and also a certain effect of HM length. Liposome composition PE : PG resembling G^−^ membranes showed to be more prone to LEGO-LPPO-induced lysis than those resembling G^+^ membranes. All the tested molecules (at 1 mg L^−1^) caused the leakage of ∼20–60% of the LUV content in case of PE : PG membranes in contrast to ∼5% in case of both PG : PE and PG : CL membranes. The C7 with shortest HM induced the lowest leakage in all types of the membranes. Interestingly, leakage of G^−^ resembling LUV (PE : PG) exhibits biphasic behavior, which we already noted *in vivo* on *E. coli* 3954 and *E. coli* imp4213 (Fig. 3). Biphasic behavior was not observed on G^+^ resembling LUVs (Fig. 7) nor *in vivo* on *S. aureus* and *B. subtilis* (Fig. 5).

Next, we tested the dose–response of LEGO-LPPO on LUV using the range of concentrations 0.3–5 mg L^−1^ for liposomes resembling G^−^ bacteria and 0.5–12.5 mg L^−1^ for liposomes resembling G^+^ bacteria (ESI Fig. S4 and S5†).

**Fig. 8:**
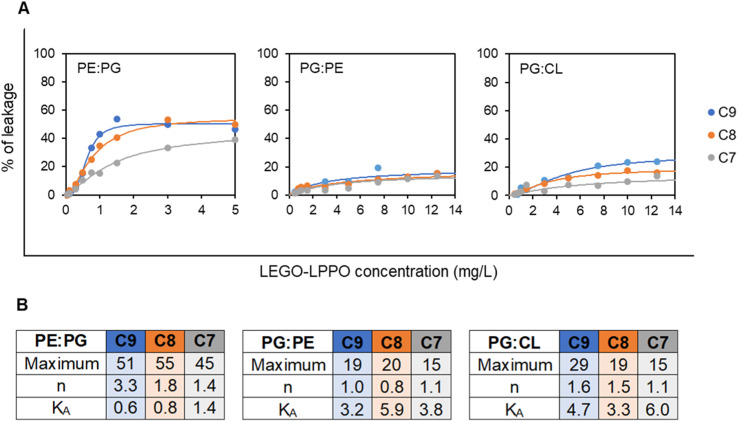
(A) Concentration dependency of leakage (after 15 min of incubation with LEGO-LPPO) of CF-loaded LUV_1000_ of various composition – 2 : 1 (w/w) mixtures – PE : PG (mimicking *E. coli*), PG : PE (mimicking *B. subtilis*) and PG : CL (mimicking *S. aureus*). Note that for PE : PG liposomes the used LEGO-LPPO concentration range was 0.5–5 mg L^−1^, whereas for PG : PE and PG : CL the 0.5–12.5 mg L^−1^ range was applied. The curves show the best fits of Hill function. (B) The parameters of the fitted Hill functions: maximum signifies the highest reachable liposome leakage, *n* is the Hill number and *K*_A_ is the LEGO-LPPO concentration inducing half-maximal leakage (see Materials and methods for details).

Generally, the liposomes were disrupted to different final extents, depending on LUV composition, the LEGO-LPPO dosage and the length of its HM. Again, LEGO-LPPOs were most active on PE : PG LUVs with *K*_A_ (concentration inducing half-maximal leakage) in range 0.6–1.4 mg L^−1^ for all tested molecules (Fig. 8). On the other hand, *K*_A_ values were significantly higher (in range 3.2–6.0 mg L^−1^) on PG : PE and PG : CL liposomes resembling G^+^ membranes. Overall, LEGO-LPPO were effective in disrupting LUV membranes in order C9 > C8 > C7. However, such dependency was not observed on PG : PE membranes (Fig. 8).

Interestingly, on PE : PG LUVs with 67% content of PE we observed relatively highly cooperative action of LEGO-LPPO with Hill numbers *n* about 3.3, 1.8 and 1.4 for C9, C8 and C7, respectively. On the liposomes with lower PE content of 33% (PG : PE) LEGO-LPPO showed no cooperativity with *n* values about 1. On the LUVs composed of PG : CL LEGO-LPPOs caused moderate cooperativity, *n* ∼ 1.5 (Fig. 8).

## Discussion

When considering the properties of any newly designed antimicrobial molecules we largely deal with their hydrophobicity. Hydrophobic groups are needed for effective membrane insertion of the molecules. In this research, we compared the difference in the overall activity of LEGO-LPPO molecules differing in the length of the alkenyl hydrophobic module by one carbon atom (Fig. 2). Notably, with the MIC ∼1–3 mg L^−1^ and HC_50_ > 200 mg L^−1^, LEGO-LPPOs possess remarkable antimicrobial activity and low hemolytic activity (Table 1). Our data show that the effectiveness of LEGO-LPPOs in terms of MIC is influenced by length of the HM – elongation of the alkenyl chain from 7 up to 9 carbon atoms increases antimicrobial potential. In moderately sensitive bacteria (*E. coli* CCM 3954 and *S. aureus*) the lengthening of the HM by one carbon atom resulted in halving of the MIC value. However, increasing the alkenyl chain length up 10 carbon atoms resulted in drop of the antimicrobial activity and increased hemolytic activity at the same time. We concluded that C7, C8 and C9 are within an optimum hydrophobicity window in which high antimicrobial activity and selectivity could be obtained. These LEGO-LPPOs were selected for the subsequent structure–activity relationship study. We studied the effects of a wide range of LEGO-LPPO concentration *in vivo* and *in vitro*. Even though selected molecules (C7, C8, C9) were effective on both G^+^ and G^−^ bacteria, we observed significant differences in overall LEGO-LPPO performance.

Our *in vivo* experiments with PI imply that LEGO-LPPO may have different action mechanisms against G^+^ and G^−^ bacteria. While the membrane permeabilization of *S. aureus* and *B. subtilis* was immediate (Fig. 5), biphasic action was observed for *E. coli* 3954 (Fig. 3A–C). The first phase of permeabilization, dominated in the case of C9, was still present with C8 but almost vanished with C7. The second postponed phase of permeabilization was comparable in all tested molecules with rate ∼0.5% min^−1^ (Fig. 4). Note that we analyzed those concentrations that induced comparable membrane perforation. Interestingly, the biphasic behavior of membrane permeabilization (sigmoidal kinetics) was never observed for G^+^ bacteria. At high LEGO-LPPO concentration the membrane permeabilization of *S. aureus* and *B. subtilis* is very fast, yet clearly dose-dependent. We cannot rule out that the detailed mechanism of disruption of the cytoplasmic membrane in G^+^ and G^−^ bacteria is different. A minor modification in the membrane composition and/or structure of the antimicrobial agent can lead to different antimicrobial mechanisms resulting in the membrane damage.^33,34^ It is possible that in the case of G^+^ bacteria there occurs a local rupture of the membrane or even its gradual micellization by LEGO-LPPO.

It seems that in the case of *E. coli* 3954 the OM creates an obstacle that needs to be eliminated before LEGO-LPPO can reach the cytoplasmic membrane. Off note, C9, C8 and C7 molecules differ in perforation potential against *E. coli* 3954 with intact OM, while C9 is the most active and C7 the least active. On the other hand, this does not apply for *E. coli* imp4213 strain (bearing permeability defects of OM) where all the molecules probably reached the cytoplasmic membrane directly and showed almost identical permeabilization kinetics (Fig. 3D–F). From these results we deduce that in *E. coli* the cytoplasmic membrane itself is probably permeabilized in a similar way by C9, C8 and C7. When tested on *E. coli* imp4213, the LEGO-LPPO exerted activity resembling closely the situation on both G^+^ bacteria where the OM is absent naturally. We propose that the reason for the lowest antimicrobial activity of C7 against *E. coli* is its inability to overcome the outer membrane which we observed for the C6 molecule bearing a saturated alkyl chain (not shown). Similar effect of OM was described for molecules from the previous generation of LPPO I which were also unable to overcome the outer membrane and were ineffective against G^−^ bacteria but they exerted an antimicrobial action against *E. coli* imp4213.^20^ We speculated that LEGO-LPPO oligomers with different number of subunits may form the active pore in time. The different populations of LEGO-LPPO oligomer pores might be represented by the distinct phases of PI influx. Possibly, to fully disrupt cytoplasmic membrane of G^+^ and G^−^ bacteria, diverse LEGO-LPPO oligomers might take place depending on the target membrane composition.

To test this hypothesis, we conducted electro-physiological experiments on planar lipid membranes composed of *E. coli* polar lipids and *n*-decane. We observed a broad range of single-pore conductances (from few pS up to 2000 pS) dependent on treatment concentration (ESI Fig. S6†). Generally, the higher LEGO-LPPO doses induced formation of pores with higher conductance more frequently, usually with unpredictable dynamics. Varying conductances of LEGO-LPPOs pores observed at different tested concentrations suggest the occurrence of manifold pore stoichiometry with diverse pore geometry. We should admit that the fast pore dynamic cannot be detected properly due to limited time resolution of the instrument. However, we can conclude that the tested LEGO-LPPOs were able to form dynamic pores permeable for K^+^ and Cl^−^ ions.

The average pore properties of C9 were also tested on liposomes composed of PE : PG 2 : 1 resembling phospholipids of *E. coli* membrane (ESI Fig. S7†). Liposomes filled with fluorophore–quencher pair ANTS^−^/DPX^+^ exerted continuous leakage of both probe molecules when treated with C9. However, DPX^+^ leaked about two-times faster than ANTS^−^ for tested C9 concentration range 0.1–6 mg L^−1^. This result suggests that molecules of molecular weight ∼400 g mol^−1^ (*i.e.* molecular weight of the probes) can pass C9 pores with slight preference for positively charged ones.

Next, we asked whether the delayed permeabilization of G^−^ bacteria with biphasic kinetics (Fig. 3 and 4) might be caused by gradual formation of larger LEGO-LPPO oligomers induced by a specific composition of their cytoplasmic membranes. For that purpose, we used synthetic phospholipid membrane vesicles, roughly mimicking the cytoplasmic membrane composition of bacteria under the study. We are aware that, for example, the increased sensitivity of a particular bacterium to LEGO-LPPO does not translate directly into increased lysis of liposomes of the corresponding composition, which was also the case of both phospholipid compositions resembling G^+^ bacteria. At odds with the MIC, these LUVs were less sensitive than the G^−^ one. It is clear from this that the differences in the sensitivity of bacteria are far from caused only by the composition of their membranes. Nevertheless, we decided to study the role of specific phospholipids on LEGO-LPPO activity. We observed that *in vitro* experiments on LUVs showed a comparable dependence of activity on HM length as *in vivo* experiments.

Experiments conducted on liposomes filled with CF showed that composition PE : PG (resembling G^−^ cytoplasmic membrane of *E. coli*) is more prone to membrane perforation with maximum of ∼70% content leakage after the treatment with C9 (Fig. 8 and S4A–C†). High dosages of all three LEGO-LPPOs induced biphasic, slightly sigmoidal character of the leakage kinetics on PE : PG liposomes and sigmoidal concentration dependency, indicating cooperative behavior of the molecules.^35^ Since the biphasic leakage character was not observed at low concentrations (0.5–0.75 mg L^−1^), we can assume that there are two different modes of LEGO-LPPO action. It is clearly most probable that decreasing the LEGO-LPPOs concentrations below a certain threshold decreases the probability of intermolecular interaction and oligomer formation.^36^ Therefore, large pores might be less frequent or completely missing, and the kinetics no longer shows sigmoidal character. We would like to emphasize that the biphasic leakage of LUVs is not linked generally to membranes of high PE content. We already observed more complex leakage kinetics in various membrane compositions treated with lipopeptide surfactin.^37^

We used the Hill function to fit dose–response data showing percentage of leakage as a function of LEGO-LPPO concentration (Fig. 8A). The observed high Hill numbers suggest that C9 exhibits a more cooperative behavior than the other two molecules on LUV composition mimicking G^−^ bacteria (PE : PG) (Fig. 8B). On the other hand, liposomes resembling roughly the *S. aureus* and *B. subtilis* membrane lipid compositions (PG : CL and PG : PE, respectively) presented lower susceptibility to LEGO-LPPO action (Fig. 8 and S4D–I†). The CF leakage reached approximately 40% and 30% for PG : CL and PG : PE, respectively. We observed only non-sigmoidal kinetics which indicate the non-cooperative behavior (ESI Fig. S4D–I†). Any increase of the treatment concentration up to 12.5 mg L^−1^ did not show dramatic rise in effectivity of the tested molecules (ESI Fig. S5†). In some cases, we observed a phenomenon of lower effectiveness after addition of the high doses of antibiotics (3 and 5 mg L^−1^ for C8 and C9). This suggests that there may be a threshold concentration above which inactive complexes are formed. As the critical micellar concentrations (CMC) are similar for all tested molecules (∼1.5 mM, not shown), we do not expect dramatic differences in their behavior in the solution. Most probably, non-functional complexes appear after formation of mixed phospholipids and LEGO-LPPO micelles. Another explanation of lowered LEGO-LPPO activity at the highest concentration might stem from the shape of their molecules. LEGO-LPPOs are low molecular weight compounds with strongly positive intrinsic curvature as they possess bulky polar parts. As a certain paradox, the massive incorporation of such molecules into the membrane might stabilize the lamellar phase by compensating overall membrane negative spontaneous curvature in certain types of phospholipid compositions, like those containing PE.^38^

Primary interaction of antimicrobials with the membrane surface is driven by electrostatic interactions followed by insertion into the membrane.^7^ Since PG : PE lipid composition holds 67% of the negatively charged PG and PG : CL lipid composition also bears an overall negative net charge, we expected higher affinity of positively charged LEGO-LPPOs to these types of membranes and therefore more effective pore formation and leakage. However, our hypothesis that LEGO-LPPOs will be most active on overall more electronegative membranes was not confirmed. In the model membrane system of liposomes (lacking the cell wall and OM of bacterial cells) we observed a dramatic effect of PE content on LEGO-LPPO leakage abilities. Namely, high content of PE (67% in PE : PG liposomes) allowed highly effective and cooperative liposome leakage by contrast to PG : PE (containing only 33% of PE) and PG : CL liposomes (Fig. 8). In addition to causing negative membrane curvature, PE also differs from PG in its ability to form H-bonds. We expect that PE head group could constitute qualitatively different and distinct complexes with amino groups of LEGO-LPPO polar module than that of PG or diphosphatidylglycerol, *i.e.* cardiolipin.^39^ On the other hand, it has been reported more generally that the high content of lipids with negative spontaneous curvature promotes membrane-disruption activity of AMPs and SAMPs.^40–42^ These observations suggest that in LEGO-LPPO-membrane interaction the phospholipid class and structure are more important in determining membrane susceptibility than the lipid headgroup charge.

**Fig. 9:**
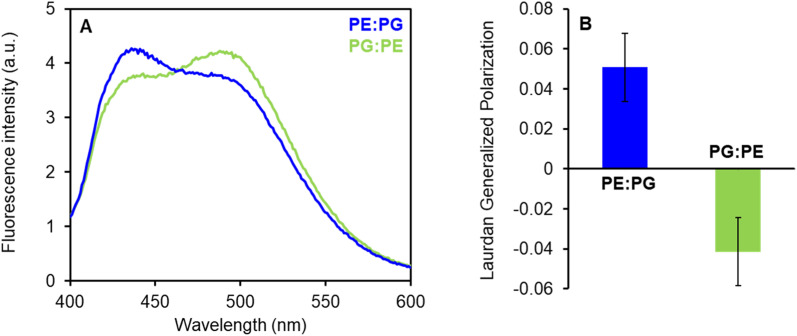
(A) Fluorescence emission spectra of Laurdan in PE : PG and PG : PE (2 : 1) membranes. The suspension of LUV_100_ (100 μM) was labeled with Laurdan (0.2 μM) at 37 °C for 60 min. Fluorescence spectra at 27 °C were measured after excitation at 365 nm using FluoroMax-3 spectrofluorometer. (B) Laurdan generalized polarization (GP) values were calculated from the spectra according to Parasassi *et al.* (1990).^28^ Positive GP values show denser lipid packing in PE : PG membranes with less water content in comparison to PG : PE mixture.

In addition to the mentioned differences in the properties of the PE : PG and PG : PE membranes, that might explain different susceptibility to LEGO-LPPO, we speculate that LEGO-LPPO insertion and/or oligomer formation might be preferred in one type of the membrane due to differences in lipid packing. We decided to quantify the differences in membrane packing and hydration of the PE : PG and PG : PE mixtures utilizing Laurdan fluorescence spectra that are influenced by water content in the membrane due to solvent relaxation effects (Fig. 9).

The fluorescence probe Laurdan exerts blue emission (∼440 nm) in the absence of water molecules but the green emission (∼500 nm) in a hydrated environment. These results show that PE : PG membranes (with GP +0.05) are considerably better packed (less hydrated) than PG : PE bilayers (GP −0.04) with lower PE content. We expect that more ordered PE : PG membranes with packed fatty acyl chains could induce certain lateral separation of inserted LEGO-LPPO molecules into distinct clusters. Such a process might further stimulate formation of LEGO-LPPO larger complexes (oligomers) responsible for membrane perforation. Comparable effects were already observed in the literature.^43–46^

Our long-term goal is to find more general rules that could be a guide in the development of new antibacterial agents acting on the bacterial cytoplasmic membrane. The current aim was to explore structure–activity relationships of LEGO-LPPOs molecules differing specifically in the lengths of their hydrophobic modules when other parts of the molecules remained unchanged. In theory, an optimum hydrophobicity window should exist in which high antimicrobial activity as well as selectivity could be obtained. It was proven that too high hydrophobicity correlates with unwanted stronger hemolytic activity of the molecules. Highly hydrophobic molecules tend to self-associate and because of that cannot pass bacterial cell wall effectively^47^ and lose their antibacterial activity. In fact, when we tested properties of the molecule with the longest HM (C10) the antimicrobial activity did not further increase and dropped down slightly (Table 1) but the undesired hemolytic activity enhanced dramatically. On the other hand, the molecule with saturated six-carbon HM (C6) is basically inactive^23^ and unable to overcome the bacterial outer membrane (not shown). In this respect it appears that the hydrophobicity window for the presented molecules is in range C7–C9 when C6 and C10 are useless in terms of their antimicrobial applications. The HM length affects the permeabilizing potential both in living bacteria and in model membranes. In addition to the length of the hydrophobic module, the composition of the target membrane plays a substantial role in the effectiveness of LEGO-LPPOs. In our case, phosphatidylethanolamine is the key component that promotes permeabilizing potential possibly by inducing cooperative pore formation by LEGO-LPPOs.

## Conflicts of interest

We certify that this paper is an original work. All co-authors have seen and agreed with the content of the manuscript. All authors declare that they have no conflict of interest to disclose.

## Supplementary Material

RA-014-D3RA07251G-s001
