# Supplementary material for: LEGO-lipophosphonoxins: length of hydrophobic module affects permeabilizing activity in target membranes of different phospholipid composition
Source: RSC Adv. 2024 Jan 17;14(4):2745–56. doi: 10.1039/d3ra07251g (PMC10792433; doi:10.1039/d3ra07251g)
Supplement: RA-014-D3RA07251G-s001 [file RA-014-D3RA07251G-s001.pdf]

Electronic Supplementary Material (ESI) for RSC Advances.
This journal is © The Royal Society of Chemistry 2024

SUPPLEMENTARY MATERIALS

LEGO-Lipophosphonoxins: Length of Hydrophobic module affects permeabilizing activity in target membranes of different phospholipid composition

Milica Dugić[1], Hana Brzobohatá[1], Viktor Mojr[2], Tereza Dolejšová[1], Petra Lišková[1], Duy Dinh Do Pham[2,3], Dominik Rejman[2], Gabriela Mikušová[1,2], Radovan Fišer[1] *

1. Department of Genetics and Microbiology, Faculty of Science, Charles University, Viničná 5, 128 00 Prague 2, Czech Republic.

2. Institute of Organic Chemistry and Biochemistry, Czech Academy of Sciences v.v.i., Flemingovo nám. 2, 166 10 Prague 6, Czech Republic.

3. current address: University of British Columbia, 3333 University Way, Kelowna, BC V1V 1V7, Canada

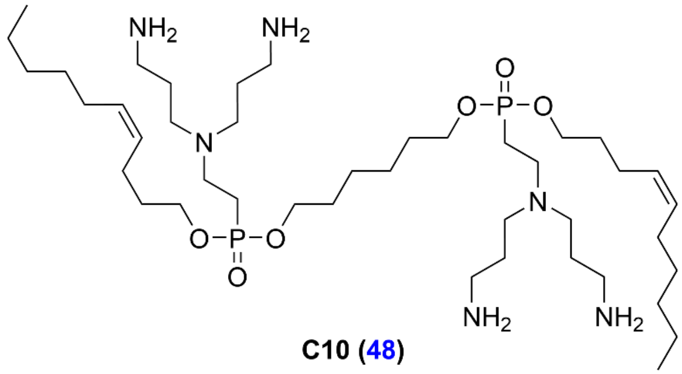

C10 (48)

Supplementary Figure S1.

Structure of C10, one of the LEGO-LPPOs selected for the study.

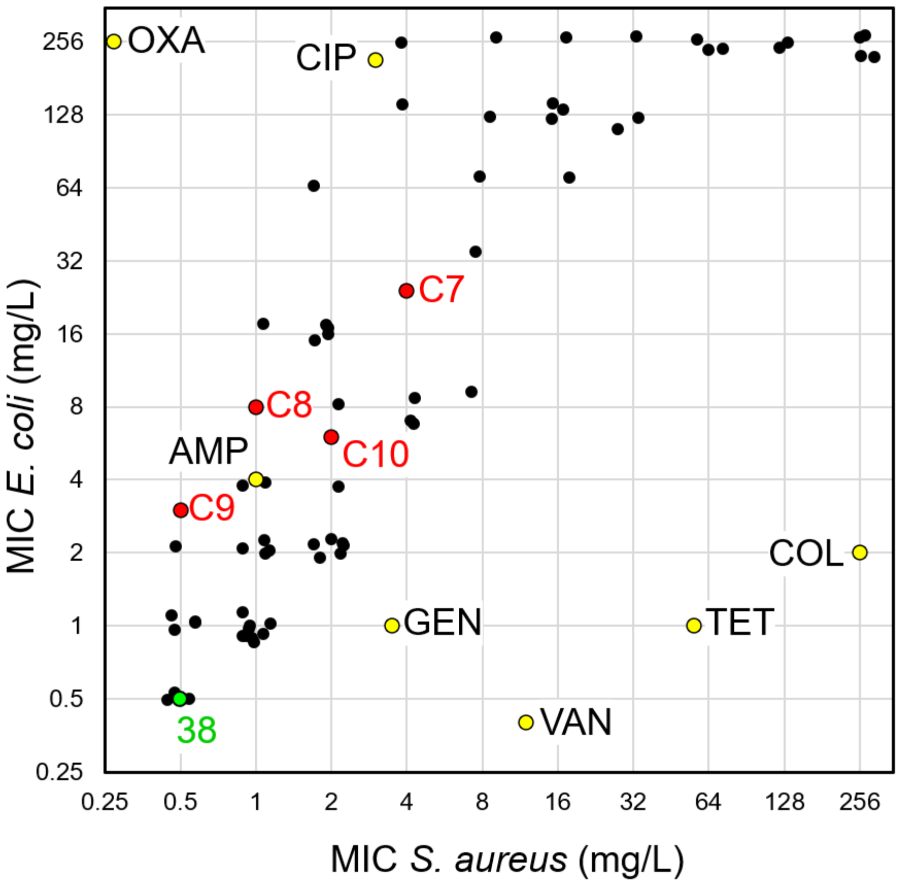

Supplementary Figure S2.

The comparison of antimicrobial activity of previously published LEGO-LPPOs and selected standard antibiotics tested on *S. aureus* and *E. coli* strains. The graph shows Minimal Inhibitory Concentration (MIC) of all LEGO-LPPO substances in publication Do Pharm et al.[1] (shown in black) with the most effective compound “38” in green. The substances that are the subject of the submitted study (C10, C9, C8, C7) are shown in red (cf. Tab. 1). Selected other commercial antibiotics are shown in yellow. Abbreviations: OXA, oxacillin; CIP, ciprofloxacin; AMP, ampicillin; COL colistin (polymyxin E); GEN, gentamicin; TET, tetracycline; VAN, vancomycin.

MIC values from literature on standard strains of *Staphylococcus aureus* CCM 4223 and *Escherichia coli* CCM 3954 were used, where available[2,3]. In other cases, median of MIC values was calculated for several bacterial strains[4,5] typically isolated from the environment. To be able to display substances with a very high MIC value (resistant strains) in the graph, the values of 256 mg/L were artificially assigned to them. Noise was artificially added to the MIC values so the points do not overlap.

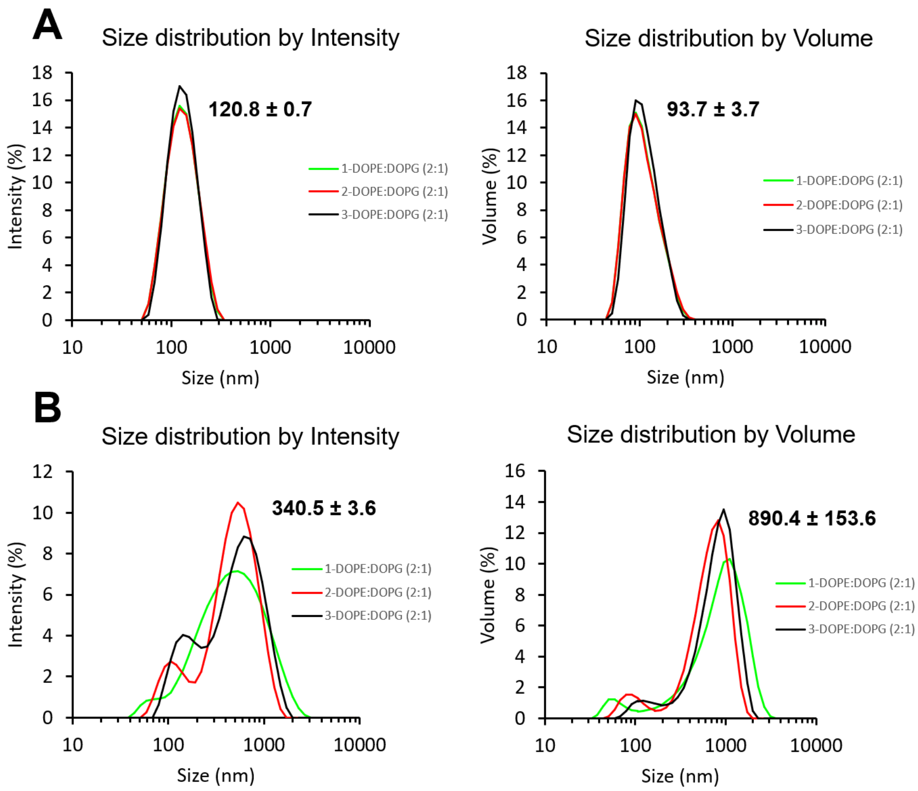

Supplementary Figure S3.

Representative dynamic light scattering (DLS) spectra for the two sizes of PE:PG liposomes (10 μM). **A)** Size distribution of LUV100 (a 100 nm filter used for extrusion) where a single peak was observed, suggesting quite narrow range of liposome diameter of about 120 nm, with polydispersity index (PDI) under 0.1 indicating reliable evaluation of the data. **B)** The size distribution analysis of the larger LUV1000 liposomes (when a 1000 nm filter was used for extrusion) showed broader distribution of intensities but still with a main significant peak around 600 nm diameter (PDI increased to about 0.347). The Z-average (340 nm) is then less reliable.

DLS measurement

Size distribution of liposomes was measured by DLS using a Zetasizer Nano ZS (Malvern Instruments Ltd., Malvern, UK) operating with a He–Ne ion laser (λ = 633 nm). The detection angle was 173 degrees. The measurements were performed in disposable polystyrene cuvettes at 25°C using buffer composed of 100 mM NaCl, 0.5 mM Na_2EDTA and 5 mM HEPES, pH 7.4. Three runs of measurements were performed for each sample. Malvern Zetasizer software was used for collecting data and their analysis. The size distribution, the hydrodynamic diameter, and the polydispersity index (PDI) were acquired from the autocorrelation fit of the data. Additionally, the volume distributions were converted from the size distributions by the software.

Supplementary Table T1.

Parameters of the Hill functions fitted to concentration dependent activity of LEGO-LPPO against *E. coli* 3954, *E. coli* imp4213, *S. aureus*, and *B. subtilis*. Membrane perforation was quantified by PI assay after 20 mins of incubation with LEGO-LPPO. The curves showing these parameters are plotted in Figure 3 and 5.

E. coli 3954	**C9**	**C8**	**C7**
Maximum	50	18	20
n	2.6	3.4	1.5
K_A	8.7	5.4	26.1

***E. coli* imp4213**	**C9**	**C8**	**C7**
Maximum	80	94	92
n	0.9	0.7	0.8
K_A	3.3	5.9	6.1

S. aureus	**C9**	**C8**	**C7**
Maximum	70	72	74
n	1.0	1.3	1.5
K_A	2.0	2.4	3.8

B. subtilis	**C9**	**C8**	**C7**
Maximum	54	50	62
n	2.0	1.9	2.0
K_A	2.4	2.3	4.8

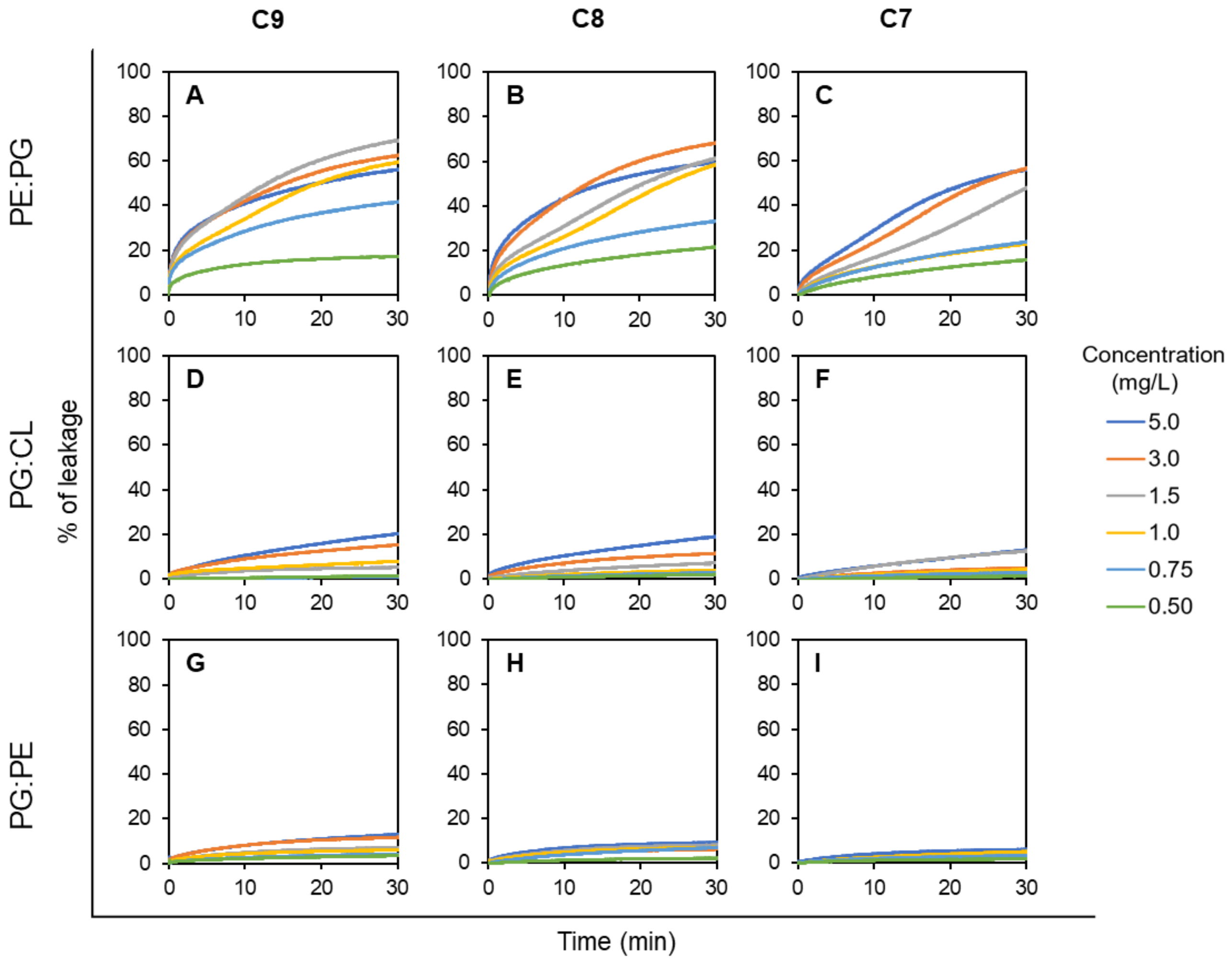

Supplementary Figure S4.

Leakage of CF from liposomes induced by LEGO-LPPOs. Curves show concentration dependent percentage of leakage from liposomes over time (30 min). Liposomes were composed of phospholipids in 2:1 ratio- PE:PG, PG:CL, PG:PE. Maximum leakage (100%) was achieved using 0.1% Triton X-100. Representative kinetics from two independent liposome preparations are shown. The data indicate that the activity of the LEGO-LPPOs depends dramatically on the liposome composition and to a certain extent on the length of the hydrophobic chain.

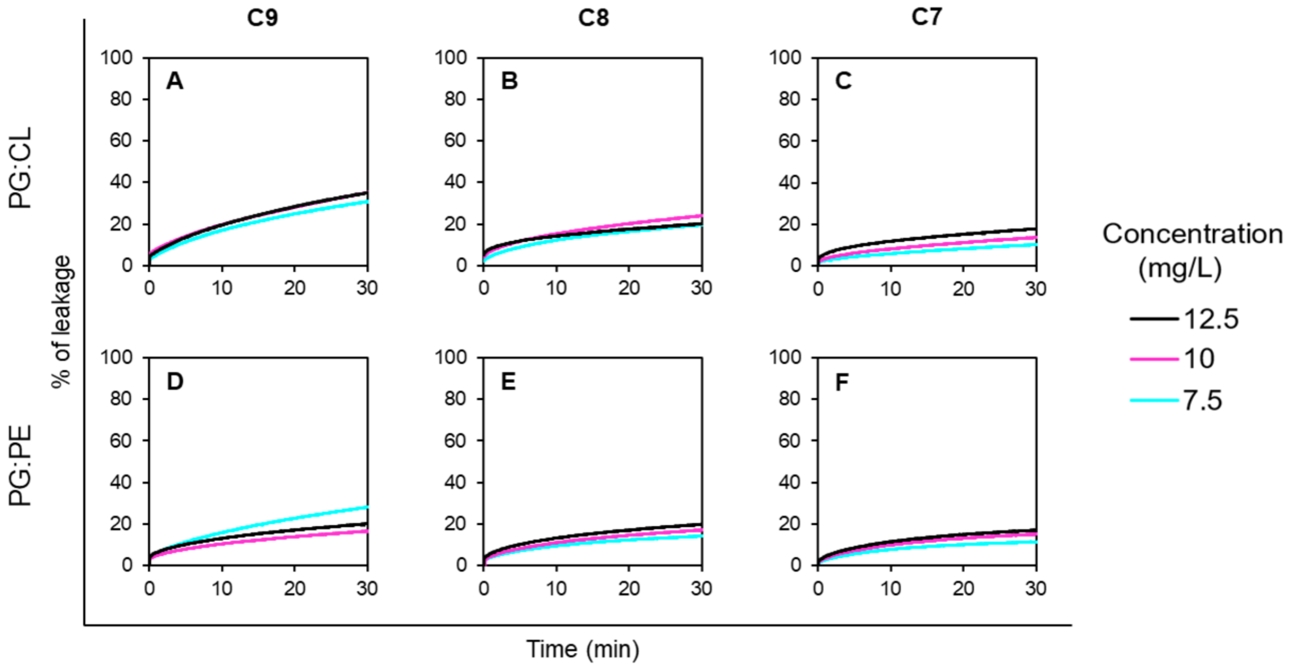

Supplementary Figure S5.

CF leakage from liposomes induced by LEGO-LPPOs. Curves show concentration dependent percentage of leakage from liposomes over time (30 minutes). Because of low effectiveness of LEGO-LPPOs on phospholipid compositions resembling G^+ bacteria (PG:CL, PG:PE), higher dosages of the LEGO-LPPOs were examined. Differences in activity between LEGO-LPPOs were observed in case of G^+ resembling compositions; C9 being the most active and C7 the least active on both compositions.

Variability of LEGO-LPPO pores in planar lipid membranes (BLM)

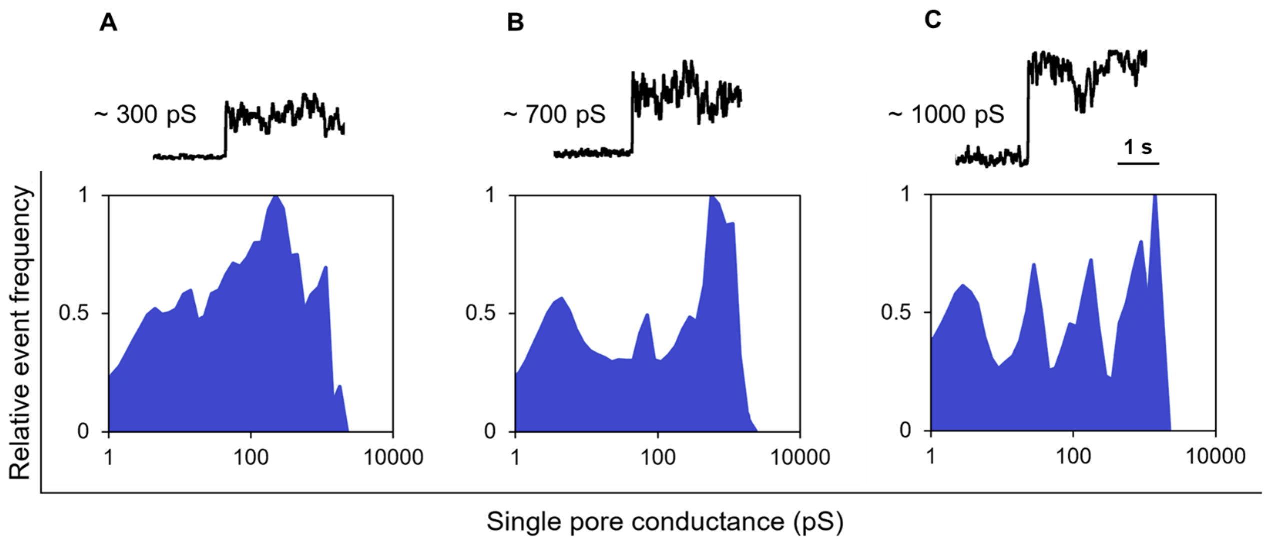

Supplementary Figure S6.

Representative single-pore recordings and cumulative whole point histograms of the electrical current of planar lipid bilayer with C9 added at different concentrations: **A)** 1.25 mg/L, **B)** 2.5 mg/L and **C)** 5 mg/L. Logarithmic histograms were constructed from ~20 s recordings on individual membranes (n>8). The membranes were composed of 3% *E. coli* lipids (*E. coli* Polar Lipid Extract, Avanti Polar Lipids) in n-decane/butanol (9:1, v/v), electrolyte contained 1 M KCl, 10 mM Tris, pH 7.4.

Membrane permeabilization assay (Figure 3) showed that all three tested LEGO LPPOs are creating pores in *E. coli* membranes. The biphasic mode of LEGO-LPPO action on the membrane suggested formation of two (or more) different populations of pores which might probably differ in pore size. Using conductivity measurements on planar lipid membranes constituted of *E. coli* phospholipids we aimed at observing individual LEGO-LPPO pores formed in the *E. coli*-derived membrane. In this experiment we used the most active compound C9 in three different treatment concentrations of 1.25, 2.5 and 5 mg/L. C9 formed a variety of pores with the single-pore conductances (Supplementary Figure S6) ranging from 10^{-12} to 2×10^{-9} Siemens. Most of the pores showed to be stable in an open state for different time intervals (from <1 second up to a few minutes), but we also observed unstable pores with variable conductance. In the concentration of 1.25 mg/L most of the pores appear

in the range up to 200 pS (Supplementary Figure S6, A). Higher concentrations (2.5 mg/L and 5 mg/L) induce the occurrence of high conductance pores: ~600 pS, 1000 pS and 1500 pS, respectively (Supplementary Figure S6, B and C). In line with the membrane permeabilization assay our results indicate that C9 molecules act in a highly cooperative manner and could form narrow pores with low conductance. However, C9 can probably assemble into large oligomers causing membrane instability of high conductance.

Planar Lipid Membrane Experiments - Method

Experiments were performed in a Teflon chamber divided into two compartments by a diaphragm with a circular aperture of approximately 0.5 mm in diameter. Planar lipid bilayers were formed across the aperture with a solution of 3% *E. coli* lipids (*E. coli* Polar Lipid Extract, Avanti Polar Lipids) in n-decane/butanol (9:1, v/v). Both chamber compartments contained 1.5 mL of 1 M KCl, 10 mM Tris, pH 7.4. The temperature was kept at 25 °C. LEGO-LPPO was added to the trans compartment of the chamber in the concentration of 1.25, 2.5 or 5.0 mg/L. Membrane current was registered with Ag/AgCl electrodes with applied membrane voltage of 50 mV (trans negative), amplified by an LCA-200-100G amplifier (Femto), and digitized by a KPCI-3108 card (Keithley). Recorded signal was processed with QuB software[6].

Mechanism of LEGO-LPPO-induced liposome perforation ($ANTS^-/DPX^+$)

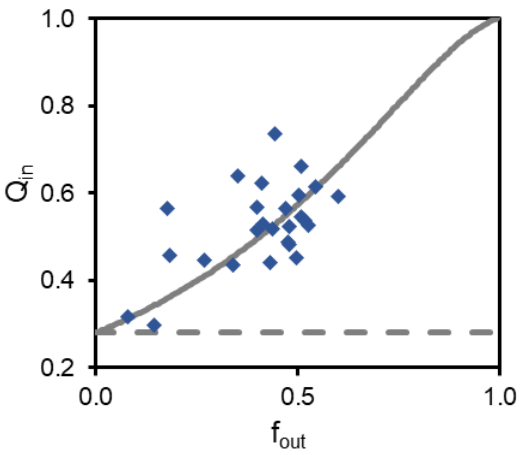

Supplementary Figure S7. Mechanism of $ANTS^-/DPX^+$ leakage from PE:PG liposomes (LUV_{100}, 10 μM phosphate content) induced by varying concentrations of C9 (in range 0.1-6 mg/L) after 30 minutes of incubation. The quenching of $ANTS^-$ by DPX^+ inside liposomes (Q_{in}) changes when fraction of $ANTS^-$ outside (f_{out}) increases which signifies the graded leakage. The solid line shows the model fit of graded DPX^+ preferential leakage (α=2). The dashed line illustrates the "all-or-none" leakage (α=0) which does not take place.

To recognize the mechanism of LEGO-LPPO membrane disruption, we introduced the liposomes loaded with fluorophore/quencher pair $ANTS^-/DPX^+$. Lipid composition of liposomes was PE:PG 2:1 (w/w) and resembled phospholipid composition of *E. coli* membrane[7]. This method can primarily be used to differentiate between two mechanisms of leakage: "all-or-none" and "graded". The "all-or-none" leakage results in two populations of vesicles. We can distinguish between "empty" vesicles, from which all the inner content leaked, and intact vesicles with unchanged concentration of $ANTS^-/DPX^+$ inside. Leakage is considered as "graded" if all the vesicles are affected to a certain extent and part of their inner content is leaking continuously from all of them. If the leakage is "graded", it is possible to distinguish the faster efflux of DPX^+ or $ANTS^-$. To easily describe the leakage selectivity, a parameter of preferential release (α) was introduced which signifies the ratio of membrane permeability for DPX^+ and $ANTS^-$[8]. The value of α=0 signifies "all-or-none" leakage, α=1

represents non-selective “graded” leakage, while the other values usually signify preferential graded leakage of DPX^+ or $ANTS^-$ molecules.

In our experiment, we tested selectivity of the most active LEGO-LPPO molecule C9 on PE:PG liposomes in a wide concentration range (0.1-6 mg/L). Our results presented in Supplementary Figure S5 show graded DPX^+ preferential leakage (α~2). Such a value is usually explained by formation of transient membrane pores that allow DPX^+ flux after its accumulation in the proximity of negatively charged membranes[8].

Mechanism of dye leakage from liposomes - Requenching Method

The requenching method was used to differentiate the mode of liposome leakage caused by LEGO-LPPOs. Briefly, using the method described in detail in Ladokhin et al., (1995)[9] one measures the dependence of $ANTS^-$ quenching inside the vesicles (Q_{in}) as a function of the fraction of $ANTS^-$ that has leaked out of the vesicles (f_{out}) after incubation with membrane-active compounds. The suspension of LUV_{100} diluted to 10 μM final phospholipid concentration and loaded with fluorophore/quencher pair $ANTS^-/DPX^+$ (2 mL in quartz cuvettes) were treated with the range of LEGO-LPPO concentrations. Tested substance (C9) was added to suspension from a diluted stock (0.1 mg/L) in large volumes (~600 μL), to avoid localized artifacts (immediate leakage induced by locally high concentration) that could affect the data interpretation. The buffer was used to compensate for the differences in volumes of individual additions. Each addition was followed by fast mixing and the samples were then incubated for an hour to reach the plateau level of fluorescence. Then, 30 μL of concentrated DPX^+ was added into the individual samples (158 mM final concentration) for determination of total quenching followed by adding 5 μL of 10% Triton X-100. Fluorescence intensities were recorded using FluoroMax-3 spectrofluorometer (Jobin Yvon, Horiba) as follows: Emission spectra (in the range of 420-600 nm, excitation at 360 nm) were measured using excitation and emission filters (365WB50 and 3RD410LP, Omega Optical) with bandwidth of 8 nm. Intensities in the range 500-510 nm were integrated for data analysis after background subtraction. Obtained intensity values were used for calculation of quenching inside of liposomes (Q_{in}), fraction of $ANTS^-$ outside (f_{out}) and a value of selectivity parameter α, according to Ladokhin et al., (1995)[9].

REFERENCES

1 D. D. Do Pham, V. Mojr, M. Helusová, G. Mikušová, R. Pohl, E. Dávidová, H. Šanderová, D. Vítovská, K. Bogdanová, R. Večeřová, M. H. Sedláková, R. Fišer, P. Sudzinová, J. Pospíšil, O. Benada, T. Křížek, A. Galandáková, M. Kolář, L. Krásný and D. Rejman, *J Med Chem*, 2022, **65**, 10045–10078.

2 Y. Gu, S. Wang, L. Huang, W. Sa, J. Li, J. Huang, M. Dai and G. Cheng, *Antibiotics*, 2020, **9**, 791.

3 S. Speck, C. Wenke, A. T. Feßler, J. Kacza, F. Geber, A. D. Scholtzek, D. Hanke, I. Eichhorn, S. Schwarz, M. Rosolowski and U. Truyen, *Heliyon*, 2020, **6**, e04070.

4 M. C. Lima, M. de Barros, T. M. Scatamburlo, R. C. Polveiro, L. K. de Castro, S. H. S. Guimarães, S. L. da Costa, M. M. da Costa and M. A. S. Moreira, *BMC Microbiol*, 2020, **20**, 127.

5 S. Ul Haq, L. Wang, W. Guo, A. I. Aqib, A. Muneer, M. Saqib, S. Ahmad, M. Ghafoor, A. Iftikhar, K. Chen and J. Liang, *Front Vet Sci*.

6 C. Nicolai and F. Sachs, *Biophys Rev Lett*, 2013, **08**, 191–211.

7 W. Dowhan, *Annu Rev Biochem*, 1997, **66**, 199–232.

8 A. S. Ladokhin, W. C. Wimley, K. Hristova and S. H. White, in *Methods in Enzymology*, 1997, vol. 278, pp. 474–486.

9 A. S. Ladokhin, W. C. Wimley and S. H. White, *Biophys J*, 1995, **69**, 1964–1971.
